# Evidences of neurological injury caused by COVID‐19 from glioma tissues and glioma organoids

**DOI:** 10.1111/cns.14822

**Published:** 2024-06-25

**Authors:** Huimin Hu, Chen Wang, Rui Tao, Bohan Liu, Dazhao Peng, Yankun Chen, Wei Zhang

**Affiliations:** ^1^ Department of Molecular Neuropathology, Beijing Neurosurgical Institute Capital Medical University Beijing China; ^2^ Department of Neurosurgery, Beijing Tiantan Hospital Capital Medical University Beijing China; ^3^ Center of Brain Tumor, Beijing Institute for Brain Disorders Beijing China; ^4^ China National Clinical Research Center for Neurological Diseases Beijing China; ^5^ Chinese Glioma Genome Atlas Network (CGGA) Beijing China

**Keywords:** COVID‐19, glioma tissues, organoid, spatial transcriptome

## Abstract

**Introduction:**

Despite the extensive neurological symptoms induced by COVID‐19 and the identification of SARS‐CoV‐2 in post‐mortem brain samples from COVID‐19 patients months after death, the precise mechanisms of SARS‐CoV‐2 invasion into the central nervous system remain unclear due to the lack of research models.

**Methods:**

We collected glioma tissue samples from glioma patients who had a recent history of COVID‐19 and examined the presence of the SARS‐CoV‐2 spike protein. Subsequently, spatial transcriptomic analyses were conducted on normal brain tissues, glioma tissues, and glioma tissues from glioma patients with recent COVID‐19 history. Additionally, single‐cell sequencing data from both glioma tissues and glioma organoids were collected and analyzed. Glioma organoids were utilized to evaluate the efficacy of potential COVID‐19 blocking agents.

**Results:**

Glioma tissues from glioma patients with recent COVID‐19 history exhibited the presence of the SARS‐CoV‐2 spike protein. Differences between glioma tissues from glioma patients who had a recent history of COVID‐19 and healthy brain tissues primarily manifested in neuronal cells. Notably, neuronal cells within glioma tissues of COVID‐19 history demonstrated heightened susceptibility to Alzheimer's disease, depression, and synaptic dysfunction, indicative of neuronal aberrations. Expressions of SARS‐CoV‐2 entry factors were confirmed in both glioma tissues and glioma organoids. Moreover, glioma organoids were susceptible to pseudo‐SARS‐CoV‐2 infection and the infections could be partly blocked by the potential COVID‐19 drugs.

**Conclusions:**

Gliomas had inherent traits that render them susceptible to SARS‐CoV‐2 infection, leading to their representability of COVID‐19 neurological symptoms. This established a biological foundation for the rationality and feasibility of utilization of glioma organoids as research and blocking drug testing model in SARS‐CoV‐2 infection within the central nervous system.

## Graphical abstract

SARS‐CoV‐2 entry factors are expressed in glioma tissue and glioma organoid. The neuronal cells within glioma tissues of COVID‐19 history demonstrated neuronal aberrations in consistent with the clinical manifestation of COVID‐19 neurological symptoms. This supports glioma organoids as a model in the research of SARS‐CoV‐2 CNS infection and drug testing.

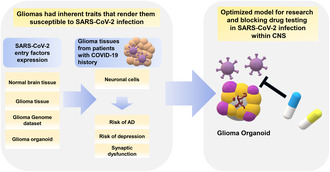

## 1 INTRODUCTION

COVID‐19, caused by the severe acute respiratory syndrome coronavirus 2 (SARS‐CoV‐2), has been reported to be associated with mild neurological symptoms such as anosmia and ageusia, as well as severe neurological symptoms including stroke and Guillain–Barre syndrome.^1^ Cognitive impairments often occur in patients experiencing long‐term COVID‐19 symptoms, commonly referred to as “brain fog,” which is one of the most frequently reported post‐infection sequelae.^2^, ^3^ There had once been a controversy regarding the potential mechanism behind the neurological symptoms associated with COVID‐19: are they consequences of the systemic manifestations of COVID‐19, or do they result from direct invasion of SARS‐CoV‐2 into the nervous system? The persistence of SARS‐CoV‐2 detected in brain at autopsy^4^ and the presence of SARS‐CoV‐2 RNA in cerebrospinal fluid provided strong evidence supporting COVID‐19 neuroinvasion and neurovirulence.^5^, ^6^ However, what are the mechanisms underlying the invasion process of SARS‐CoV‐2 into the central nervous system (CNS), and which specific cell types are susceptible to infection? Additionally, the membrane receptors that serve as direct adaptors for SARS‐CoV‐2 in brain tissue have not yet been fully revealed. Previous evidence of neurological injury caused by SARS‐CoV‐2 mainly came from direct observations of autopsy^7^ or simulated infection experiments using brain organoids as models.^7^, ^8^ Autopsies are often challenged by biosafety and ethical considerations. Additionally, autopsies have become more challenging as the lethality of COVID‐19 patients has continued to decrease.^9^ Brain organoids are exceptional models for mimicking brain development and neurological functions.^10^ However, these brain organoids are orchestrated with neuronal cells derived from induced pluripotent stem cells (iPSCs) and lack of neurovascular unit (NVU) components,^11^ which are critical for the SARS‐CoV‐2 infection process.^12^ Researchers have made efforts to incorporate NVU components by inducing choroid plexus formation in brain organoids expressing ACE2^13^ or by co‐culturing pericyte‐like cells with brain organoids.^14^ However, despite these advancements, these artificial “mini‐brains with blood vessels” still differ from natural brains in modeling SARS‐CoV‐2 entry.

Here, we collected glioma tissues derived from patients who had undergone COVID‐19 months before glioma resection surgery and performed spatial transcriptome sequencing. We proved that SARS‐CoV‐2 entry factors were expressed in glioma tissues via spatial transcriptome, single‐cell sequencing, and evidences from immunofluorescence. We then explored the impacts of COVID‐19 on neurological function. We were surprised to find that the neuronal cells in glioma tissues from glioma patients who recently experienced COVID‐19 had a remarkable tendency to Alzheimer's disease, depression, and synaptic dysfunction. The neuron remodeling was changed as well. We proposed glioma organoids as a better in‐vitro model for research of SARS‐CoV‐2 invasion in brain tissue than brain organoids. Considering the limited sources of brain tissue from patients who have undergone COVID‐19, surgically resected glioma tissues from glioma patients who have undergone COVID‐19 provide an alternative window to observe the neuronal damages caused by SARS‐CoV‐2 infection. Likewise, glioma organoids could be applicable as research and blocking drug testing model for SARS‐CoV‐2 infection within the CNS.

## 2 METHODS

### 2.1 Patients' information and tissues

Glioma tissue samples were surgically removed and obtained from the Glioma Treatment Center of Beijing Tiantan Hospital. This study received approval from the Institutional Review Boards of Beijing Tiantan Hospital, and written informed consent was obtained from all participating patients. Detailed information regarding the patients' history of COVID‐19 and their glioma resection surgeries is provided in Table S1.

### 2.2 Spatial transcriptome data acquirement and processing

The spatial transcriptome data of glioma tissues from four glioma patients who had undergone COVID‐19 (Glioma‐COVID19) were obtained using 10× Visium Spatial Gene Expression kit (https://www.10xgenomics.com/spatial‐gene‐expression). All the instructions for tissue optimization and library preparation were followed according to manufacturer's protocol. The spatial transcriptome data of normal brain tissue were from Heiland data.^15^ The spatial transcriptome data of glioma tissues were from Heiland data^15^ and GSE194329 data^16^ (detailed information in Table S1). R package SPATA2^15^ developed by Jan Kueckelhaus, Dieter‐Henrik Heiland, and Simon Frerich was used as the major tool in spatial transcriptome data processing. Transcriptome data (filtered_feature_bc_matrix) derived from the spatial transcriptome data were processed and analyzed using R package Seurat^17^ to describe the spots transcriptional characteristics in the context of ignoring spatial information. The scoring of the functional pathways was performed with the function PercentageFeatureSet in Seurat based on the geneset downloaded from Molecular Signatures Database (https://www.gsea‐msigdb.org). The significance of the differences in the functional pathways scores between each two comparing groups was analyzed with a *T*‐test using the function stat_compare_means in R package ggpubr. Functional annotations of the characteristic genes were performed with R package clusterProfiler.^18^

### 2.3 Single‐cell sequencing data acquirement and processing

Single‐cell sequencing of three glioma organoid lines was performed using 10× Genomics platform (Chromium Next GEM Chip G Single Cell Kit) according to manufacturer's protocol. Single‐cell sequencing data of glioma tissues were downloaded from the open date platform for public established by our team (6148 cells, involving in 73 regions from 14 patients, STRT‐seq platform, http://www.cgga.org.cn/).

### 2.4 GBM organoids operation, pseudovirus infection, and blocking drug testing

The method of GBM organoids establishment was modified from Song's protocol.^19^ Fresh GBM postoperative samples were transported to laboratory on ice. GBM samples then were rinsed in PBS (phosphate‐buffered saline) and the necrotic parts were removed in the dish. The tissues were placed in a precooled medium in a culture dish on ice and cut into pieces about 0.5 mm in diameter with ophthalmic forceps and scissors. After centrifugation (1000 rpm, 3 min), the fibrous tissue pieces were removed and only the clumpy tissue pieces were collected for culture. The tissue pieces obtained by centrifugation were resuspended with the culture medium containing 50% DMEM: F12 (Thermo Fisher Scientific) and 50% Neurobasal (Thermo Fisher Scientific) supplemented by 1× GlutaMax (Thermo Fisher Scientific), 1× NEAAs (Thermo Fisher Scientific), 1× PenStrep (Thermo Fisher Scientific), 1× N2 supplement (Thermo Fisher Scientific), 1× B27 without vitamin A supplement (Thermo Fisher Scientific), and 2.5 mg/mL human insulin (Sigma). The GBM organoids were horizontally‐shaking (100 rpm) cultured within a humidity incubator of 37°C and 5% CO_2_, and 90%.

In the drug blocking test, 15 glioma organoid spheres from a single organoid line with a diameter of 1.5–2 mm were divided into three groups with similar sizes on average. The testing reagents were Camostat Mesilate (Selleck, S2874), Nafamostat mesilate (Selleck, S1386), and Proxalutamide (Selleck, S9898). Forty‐eight hours before infection with pseudovirus, the three groups of organoids were pretreated with Camostat Mesilate, Nafamostat Mesilate, or Proxalutamide, respectively, at working concentrations of 10 μM. After 48 h of continuous treatment, the drug‐containing cell culture medium was replaced with a fresh drug‐free medium. Three groups were transfected with Pseudovirus‐2019‐nCOV (TSINGKE), and the number of transfected virus particles in each group was 5 × 10^5^. Twenty‐four hours later, the organoid spheres were rinsed with PBS, and fixed with polyformaldehyde. Frozen sections were produced and processed to immunofluorescence.

### 2.5 Immunofluorescence

The fresh glioma tissues or organoid spheres were immediately embedded with OCT (Sakura) on dry ice. Frozen sections of thickness of 10 μm were produced. Then the sections were fixed with 4% paraformaldehyde, and permeabilized with 0.1% Triton X‐100 in PBS. The sections were incubated with primary antibodies overnight at 4°C after blocking with 5% bovine serum albumin (BSA) in PBS for 1 h at room temperature. The primary antibodies used in this study were anti‐ACE2 antibody (Abcam, ab108252) and anti‐SARS‐CoV‐2 Spike antibody (Abcam, ab277624). The sections were washed with PBS and incubated with fluorophore‐conjugated secondary antibodies (Abcam) for 1 h at room temperature in the dark. Nuclei were counterstained with 4′,6‐diamidino‐2‐phenylindole (DAPI) for 5 min. Finally, the sections were mounted with anti‐fade mounting medium and imaged.

### 2.6 Schematic drawing

Workflow diagram was drawn with BioRender (www.biorender.com). The comparing table was made using graphic elements drawn by the authors with the software Paint 3D (Microsoft).

## 3 RESULTS

### 3.1 SARS‐CoV‐2 entry factors expressions in brain tissues, glioma tissues, and glioma tissues from glioma patients with recent history of COVID‐19

Whether the SARS‐CoV‐2 can infect the brain tissue directly remains a subject of uncertainty. The ambiguity arises primarily due to the scarcity of brain tissues for research proposes, particularly specimens that have been recently infected with SARS‐CoV‐2. To address this challenge, we adopted an alternative approach by collecting surgically removed glioma tissue samples from glioma patients who had recently undergone COVID‐19, aiming to elucidate the conditions and mechanisms underlying the SARS‐CoV‐2 invasion of the brain.

**FIGURE 1:**
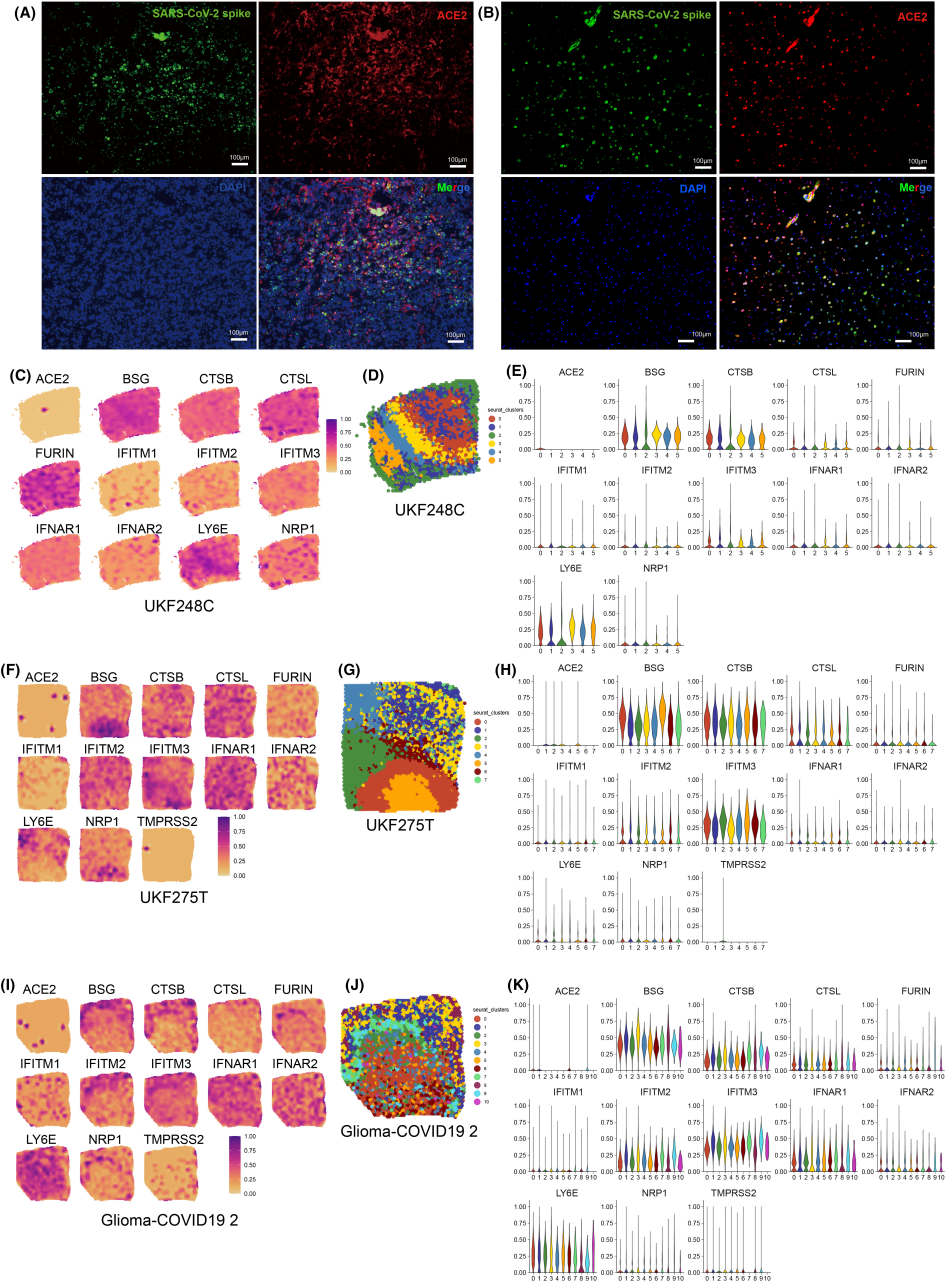
Glioma tissues are susceptible to SARS‐CoV‐2. (A) Immunofluorescent staining detecting the presence of the SARS‐CoV‐2 spike protein and ACE2 in glioblastoma tissue obtained from a patient who underwent tumor resection surgery 51 days after recovering from COVID‐19. (B) Immunofluorescent staining detecting the presence of the SARS‐CoV‐2 spike protein and ACE2 in glioma tissue (astrocytoma with *IDH* mutation, CNS WHO grade 4) from a patient who underwent tumor resection surgery 74 days after contracting COVID‐19. (C–E) Spatial expression analyses displaying the distribution of SARS‐CoV‐2 entry factors (C) in normal brain tissue (sample ID: UKF248C), including spatial cell clustering (D) and the quantity of these factors within each cell cluster (E). (F–H) Spatial expression analyses displaying the distribution of SARS‐CoV‐2 entry factors (F) in a glioblastoma tissue (sample ID: UKF275T), including spatial cell clustering (G), and the quantity of these factors within each cell cluster (H). (I–K) Spatial expression analyses displaying the distribution of SARS‐CoV‐2 entry factors (I) in a glioblastoma tissue obtained from a patient with a history of COVID‐19 (sample ID: Glioma‐COVD19 2), including spatial cell clustering (J), and the quantity of SARS‐CoV‐2 entry factors within each cell cluster (K).

A 44‐year‐old male patient who had received three doses of the COVID‐19 vaccine presented with glioblastoma in the left parietal lobe. Tumor resection surgery was performed 51 days after COVID‐19 recovery. The patient's primary complaint during COVID‐19 illness was mild body soreness lasting for 4 days. Immunofluorescence analysis revealed the presence of SARS‐CoV‐2 spike protein and ACE2 receptor expression in the resected glioma tissue (Figure 1A). In another case, a 31‐year‐old female patient who had received two doses of the COVID‐19 vaccine reported symptoms including fever, general pain, weakness, dizziness, headache, and loss of taste and smell during COVID‐19 illness. Seventy‐four days after contracting COVID‐19, surgical resection was conducted to remove an astrocytoma (*IDH*‐mutant, CNS WHO grade 4) in the right frontal lobe. Immunofluorescence analysis demonstrated the presence of SARS‐CoV‐2 spike protein and ACE2 receptor expression in the resected glioma tissue (Figure 1B).

We observed the expression of SARS‐CoV‐2 entry factors in spatial transcriptome data obtained from normal brain tissue (UKF248C from Heiland data,^15^ Table S1, Figure 1C–E), brain glioma tissue (UKF275T from Heiland data,^15^ Figure 1F–H), and glioma surgically resected samples from patients recently undergone COVID‐19 (Glioma‐COVID19 2, obtained for this study, Figure 1I–K). We found that the frequently reported SARS‐CoV‐2 entry factors were expressed in all these tissues. Notably, tissues from Glioma‐COVID19 2 exhibited a broader spectrum of SARS‐CoV‐2 entry factors expression.

### 3.2 The alterations caused by SARS‐CoV‐2 infection

**FIGURE 2:**
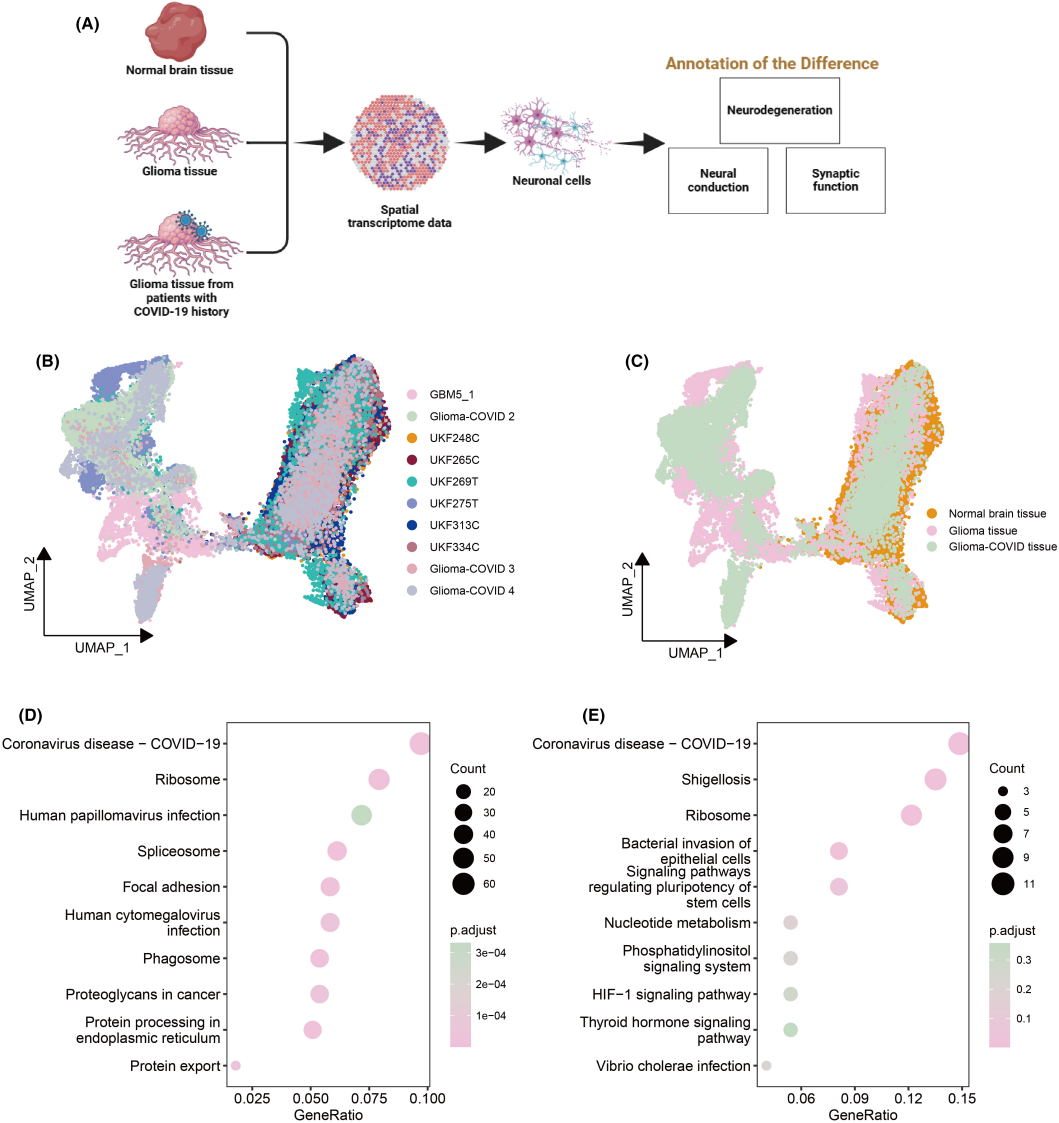
Difference analyses of normal brain tissues, glioma tissues, and glioma‐COVID tissues. (A) The workflow for the comparative analysis of the three groups of tissues. (B, C) UMAP representations depict the samples within the normal brain tissue group (comprising UKF248C, UKF265C, UKF313C, and UKF334C), the glioma tissue group (comprising GBM5_1, UKF269T, and UKF275T), and the glioma‐COVID tissue group (comprising Glioma‐COVID 2, Glioma‐COVID 3, and Glioma‐COVID 4). (D) KEGG pathway enrichment analyses of the enriched pathways with differentially expressed genes (DEGs) between glioma‐COVID tissues and normal brain tissues. (E) KEGG pathway enrichment analyses of the enriched pathways with DEGs between glioma‐COVID tissues and glioma tissues.

Glioma tissue samples obtained from glioma patients who had a recent COVID‐19 history represent invaluable research resources for examining the neuronal cell damage resulting from SARS‐CoV‐2 infection. Our objective is to elucidate the alterations, particularly the effects on neuronal cells, induced by SARS‐CoV‐2 infection through integrated analyses of normal brain tissues, glioma tissues, and glioma tissues from glioma patients with a recent COVID‐19 history (Figure 2A). Spatial transcriptome data were collected from normal brain tissues (*n* = 4, Heiland data^15^), brain glioma tissues (*n* = 4, GSE194329 data^16^ and Heiland data^15^), and glioma surgically resected samples from patients recently infected with SARS‐CoV‐2 (*n* = 4, obtained for this study). Detailed sample information including time distance between contracting COVID‐19 and glioma resection surgery, and COVID‐19 vaccine information is provided in Table S1.

Initially, we delineated neuronal cell clusters based on neuron markers (Figure S1), in conjunction with NPC2 scoring^20^ (Figure S2). One sample in the brain glioma tissue group (Glioma tissue 1) was excluded from analysis as no clearly defined cluster of neuronal cells could be discerned. Additionally, one sample in the Glioma‐COVID tissue group (Glioma tissue from patient 1 with COVID‐19 history) was excluded due to the absence of critical neuron markers in any of its cell clusters.

The included 10 samples were integrated (after de‐batching process) to illustrate their correlations and differences (Figure 2B,C). Remarkably, whether in glioma tissues or glioma‐COVID tissues, certain clusters of cells exhibited a level of health comparable to that observed in normal brain tissues. Meanwhile, a substantial number of cells in both the glioma tissue group and glioma‐COVID tissue group displayed distinct characteristics when compared with cells in normal brain tissues.

The distinct genes (differentially expressed genes, DEGs) expressed in glioma‐COVID tissues were annotated with KEGG pathway analysis. As anticipated, the most pronounced pathways enriched in glioma‐COVID tissues comparing with normal brain tissues (Figure 2D) or glioma tissues (Figure 2E) were both the COVID‐19‐related pathway.

**FIGURE 3:**
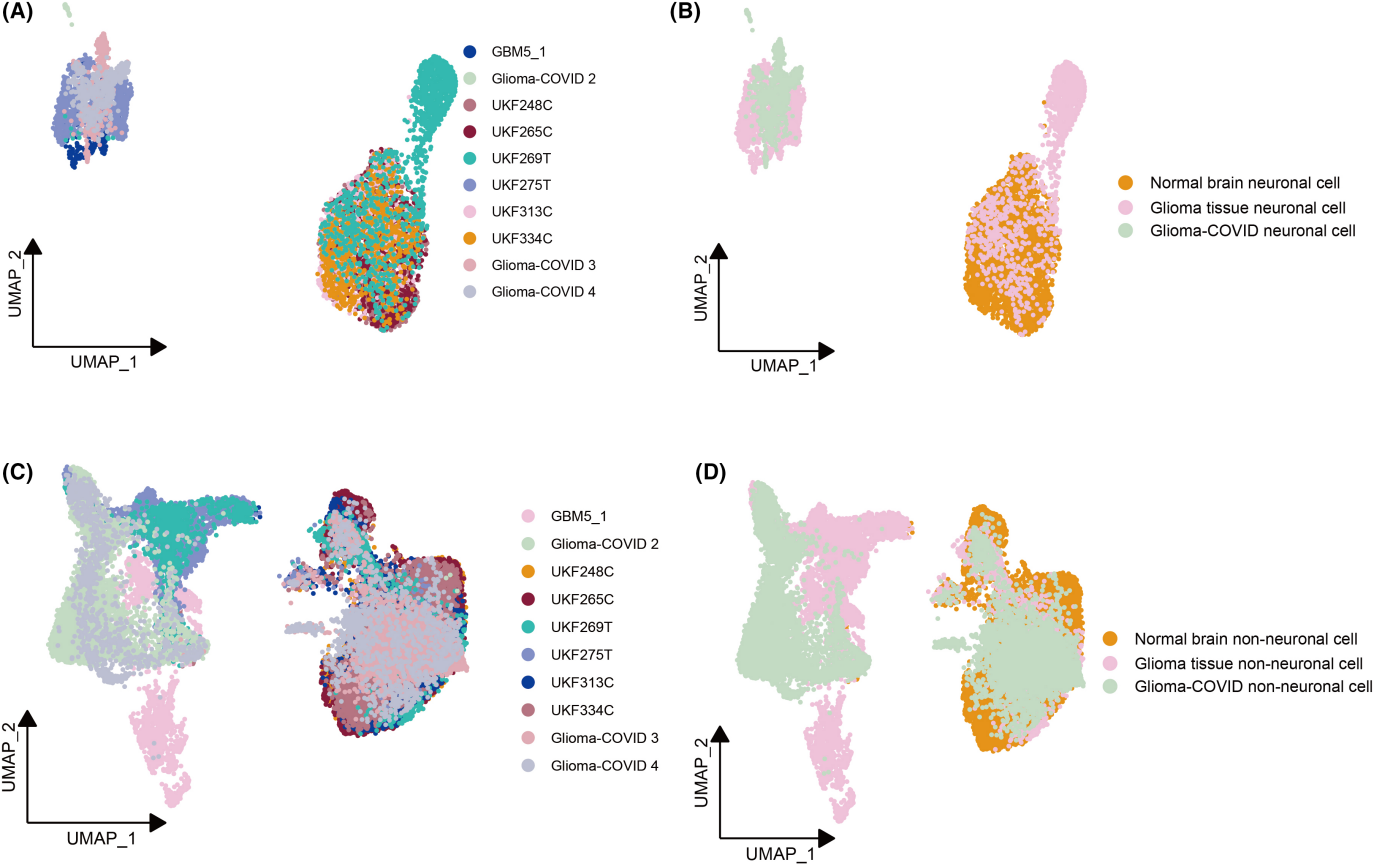
The global comparison of the differences of the neuronal cells or non‐neuronal cells in normal brain tissues, glioma tissues, and glioma‐COVID tissues. (A, B) UMAP representation of the differences of the neuronal cells in normal brain tissues, glioma tissues, and glioma‐COVID tissues. (C, D) UMAP representation of the differences in the non‐neuronal cells of normal brain tissues, glioma tissues, and glioma‐COVID tissues.

We isolated neuronal cells from each sample based on neuron markers (Figure S1), in conjunction with NPC2 scoring (Figure S2), and subsequently examined the distinctions among neuronal cells derived from the three different tissue types. Notably, neuronal cells derived from glioma‐COVID tissues exhibited a distinct separation from those from normal brain tissues (Figure 3A,B). Interestingly, in contrast to neuronal cells, non‐neuronal cells (i.e., cells other than neuronal cells) appeared to be less influenced by COVID‐19. A considerable number of non‐neuronal cells in glioma‐COVID tissues maintained a phenotype similar to that observed in non‐neuronal cells from normal brain tissues (Figure 3C,D).

**FIGURE 4:**
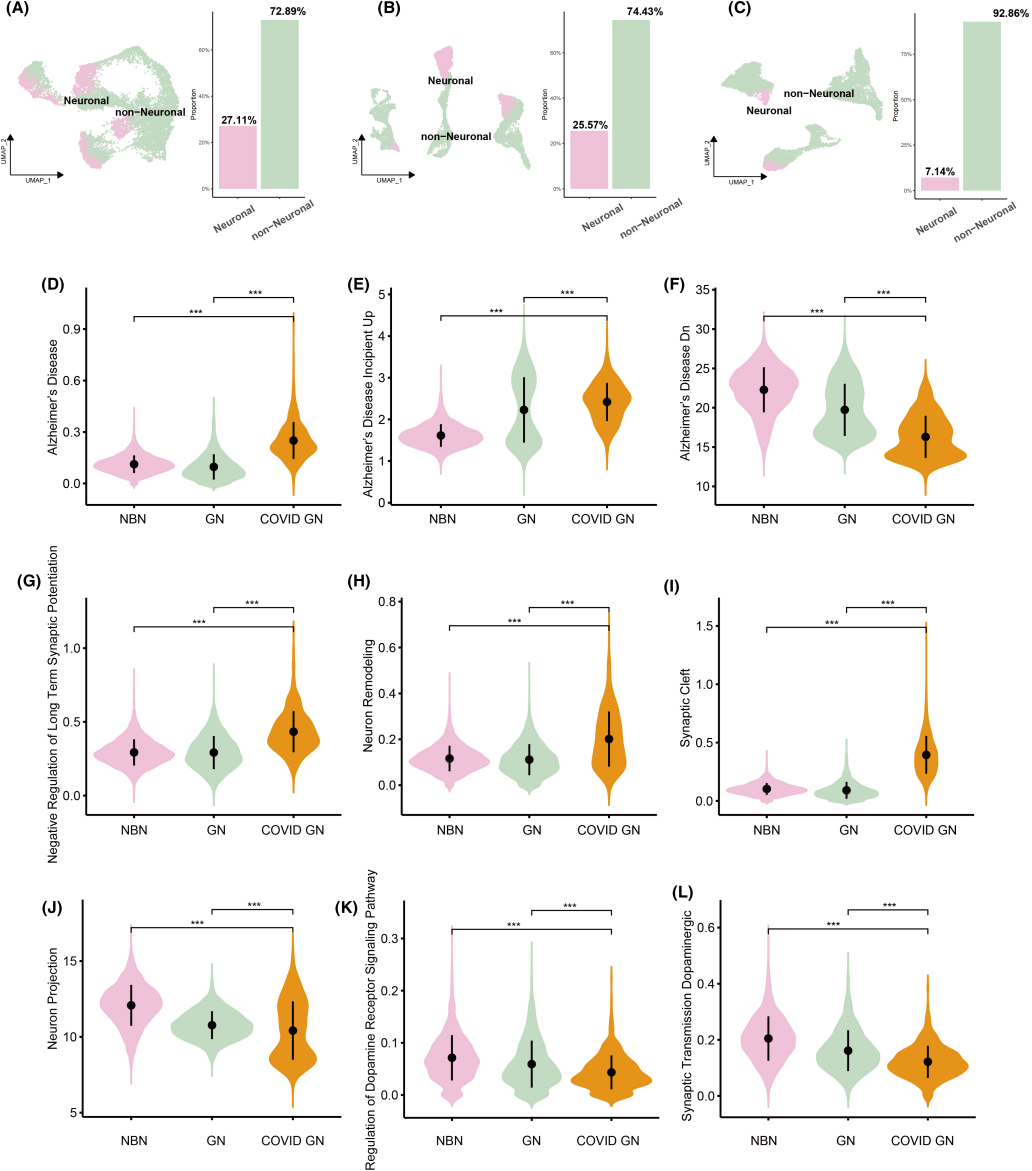
Characteristic differences between the neuronal cells in normal brain tissues, glioma tissues, and glioma‐COVID tissues. (A–C) The proportions of neuronal cells in normal brain tissues (A), glioma tissues (B), and glioma‐COVID tissues (C). (D–L) The neurological function pathways scoring of the neuronal cells in normal brain tissues (NBN), glioma tissues (GN), and glioma‐COVID tissues (COVID GN). *T*‐test was performed between the indicated two groups. *** indicates *p* ≤ 0.001.

The proportions of neuronal cells in each tissue type were depicted in Figure 4A–C. Information regarding the principal neuronal functional pathways was obtained from the GSEA Molecular Signatures Database. Gene enrichment scoring of pathways associated with Alzheimer's disease was significantly increased in glioma‐COVID neuronal cells (COVID GN), comparing with normal brain neuronal cells (NBN) or glioma neuronal cells (GN) (Figure 4D–F). Neuronal damage or dysfunction in COVID GN was suggested by increases in negative regulation of long‐term synaptic potentiation (Figure 4G), the neuron remodeling pathway (Figure 4H), and synaptic cleft pathway scoring (Figure 4I), as well as decreases in neuron projection (Figure 4J). Psychiatric disorders, including psychotic, mood, anxiety, alcohol use, and sleep disorders, have been frequently reported following COVID‐19.^21^ SARS‐CoV‐2 infection has been implicated in dopaminergic neuron damage, such as premature senescence.^22^ We observed dopaminergic neuron dysfunctions, including compromised regulation of the dopamine receptor signaling pathway (Figure 4K) and synaptic transmission dopaminergic (Figure 4L). GSEA pathway enrichment analyses provided consistent evidences (Figure S3). The genes brain expressed X‐Linked 1 (*BEX1*), brain expressed X‐Linked 3 (*BEX3*), and Stathmin 1 (*STMN1*) had been confirmed uniquely down‐regulated in excitatory‐ and inhibitory‐neuron in brain bearing AD according to single‐cell transcriptomic analysis of Alzheimer's disease.^23^ We found these three genes were expressed in a remarkable lower level in neuronal cells from glioma‐COVID tissues (COVID GN) than the neuronal cells from normal brain tissues (NBN) or the neuronal cells from glioma tissues (GN) (Figure S4).

### 3.3 Glioma organoid as an alternative in‐vitro model in research of neurotropism of SARS‐CoV‐2 and drug screening

**FIGURE 5:**
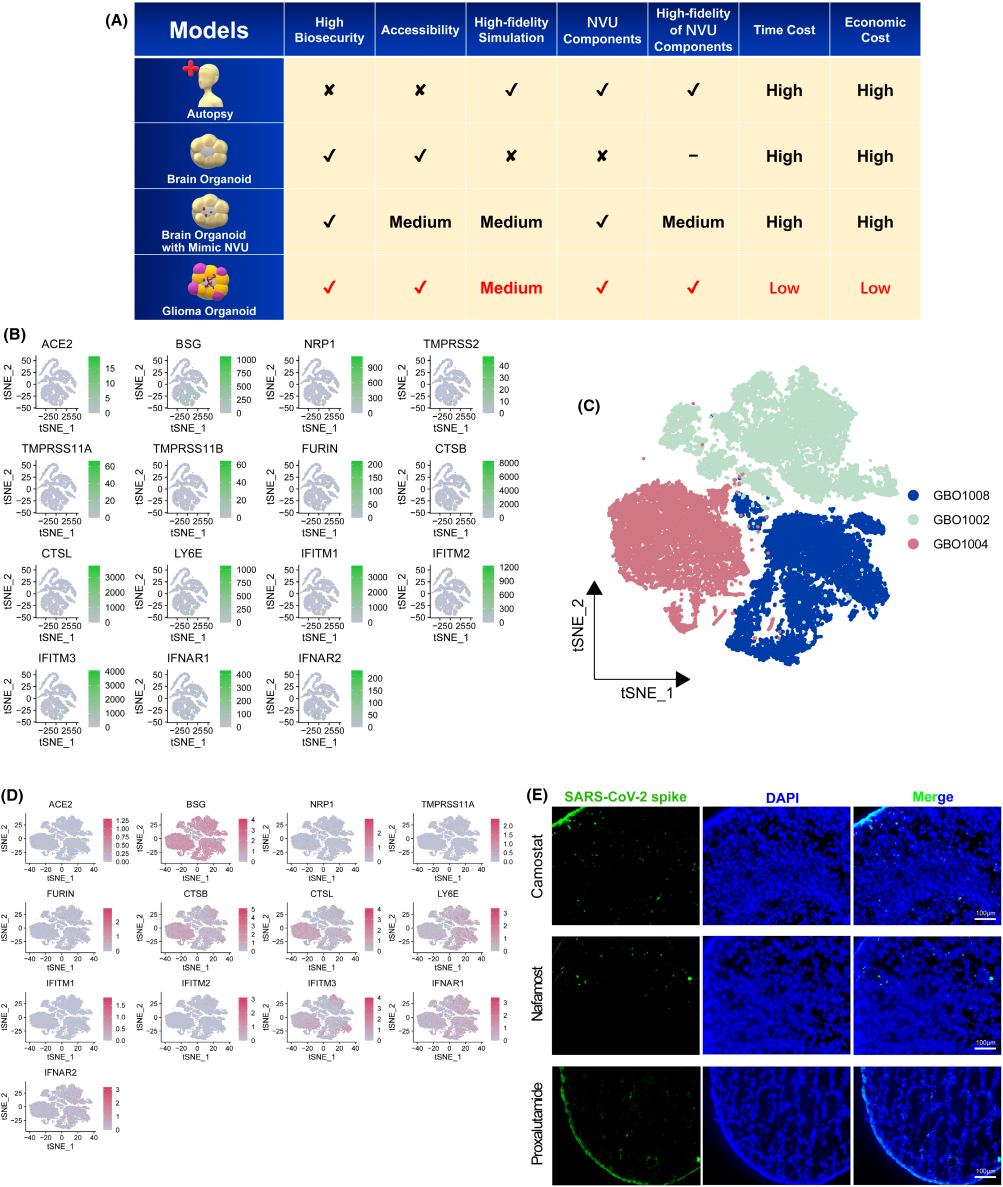
The biological foundation of the feasibility of glioma organoids as research model of SARS‐CoV‐2 invasion to the CNS. (A) The comparing chart of the advantages and disadvantages of the research models of SARS‐CoV‐2 invasion to the CNS. (B) SARS‐CoV‐2 entry factors expressions in glioma single‐cell data of CGGA. (C) TSNE representation of three lines of glioma organoids. (D) SARS‐CoV‐2 entry factors expressions in the lines of glioma organoids in C. (E) Representative staining of SARS‐CoV‐2 spike protein in the test of the blocking effects of Camostat mesylate (Camostat), Nafamost mesylate (Nafamost), and Proxalutamide in SARS‐CoV‐2 infection of glioma organoids.

The lack of ideal models to simulate the processes and mechanisms of CNS invasion by SARS‐CoV‐2 infection has contributed significantly to the obscurity of the mechanisms underlying neurological injury caused by COVID‐19. We listed the commonly utilized research models in exploring the CNS invasion of SARS‐CoV‐2, including post‐mortem brain autopsy of COVID‐19 patients,^24^ brain organoids derived from iPSCs,^25^, ^26^ and brain organoids supplemented with induced NVU components,^13^, ^14^ and compared them with glioma organoids in terms of biosecurity, accessibility, fidelity in simulating cerebral cell types and vascular components, as well as time and economic costs (Figure 5A).

Upon comprehensive consideration, we proposed that glioma organoids offer superior biosecurity and accessibility, as well as reduced time and economic costs in their preparation and operation. The most notable advantage of glioma organoids is their naturally rich of NVU components, which is particularly attributed that angiogenesis is a hallmark characteristic of gliomas and the NVU components are critical for susceptibility to SARS‐CoV‐2 infection.

We obtained glioma organoids from freshly resected gliomas following neurosurgical procedures. To further demonstrate the susceptibility of glioma organoids to SARS‐CoV‐2, we investigated the expression of SARS‐CoV‐2 entry factors based on single‐cell sequencing data of glioma tissues available in our online dataset, the Chinese Glioma Genome Atlas (CGGA, http://www.cgga.org.cn/). Notably, all the SARS‐CoV‐2 entry factors^27^ involved in the successive steps of viral docking (*ACE*, *NRP1*, and *BSG*), processing (*CTSL*, *TMPRSS11A*, *TMPRSS11B*, *TMPRSS2*, *FURIN*, and *CTSB*), and viral defense mechanisms (*IFNAR1*, *IFNAR2*, *IFITM1*, *IFITM2*, *IFITM3*, and *LY6E*) were found to be expressed in glioma tissues (Figure 5B).

To assess whether the expression of SARS‐CoV‐2 entry factors could be maintained when freshly resected glioma tissues are made into organoids, glioma organoid lines from three glioma patients were collected for single‐cell sequencing. Most of the SARS‐CoV‐2 entry factors were detected to be expressed in these glioma organoid lines (Figure 5D) supporting the concept that glioma organoids could serve as research and blocking drug testing model in SARS‐CoV‐2 infection within the CNS.

We selected three prominent drugs for COVID‐19 treatment, namely Camostat mesilate, Nafamostat mesilate, and Proxalutamide. Glioma organoids pre‐treated with these drugs were subsequently infected with SARS‐CoV‐2 pseudovirus. Unfortunately, our results indicated that none of these three drugs were able to completely block the infection of glioma organoids by the SARS‐CoV‐2 pseudovirus (Figure 5E).

## 4 DISCUSSION

We collected brain glioma tissue samples from two patients who had a history of COVID‐19 more than 50 days prior to undergoing glioma resection surgery and confirmed the presence of the SARS‐CoV‐2 spike protein within. These findings are congruent with prior reports documenting a widespread distribution of SARS‐CoV‐2 within brain tissue samples obtained during post‐mortem examinations conducted 7 months following acute COVID‐19 infection.^4^ Furthermore, these observations lend theoretical support to the manifestation of associated neurological symptoms in cases of long COVID.^28^

Our research demonstrated the presence of cell populations within glioma tissue that closely resemble normal brain cells. These cells hold significant research implications due to the lack of available in‐vitro models representing normal brain for studying SARS‐CoV‐2 invasion of the CNS. We investigated the expression of SARS‐CoV‐2 entry factors in glioma organoids and found minimal differences compared to glioma tissue, indicating that the cultured glioma organoids largely retained the characteristics of glioma tissue and could serve as a model for studying SARS‐CoV‐2 neurotropism, as well as for screening and testing potential therapeutic interventions.

The utilization of brain organoids in research of neuroinfectious diseases demonstrates remarkable efficacy.^26^ Whereas, the glioma organoids have not ever been proposed as any research model other than the disease of glioma. In fact, the application of glioma organoids as bioactive model in the study of CNS infection and drug development in the context of COVID‐19 offers significant advantages by circumventing ethical and biosafety concerns associated with obtaining post‐mortem brain samples from COVID‐19 patients. Additionally, with the reduced virulence of COVID‐19 and a significant decrease in mortality rates, the accessibility of autopsy samples has markedly declined. However, the ongoing and worsening health impact of COVID‐19‐related neurological symptoms among the large population of long‐term COVID patients underscores the urgent need for alternative research models.

Therefore, the optimization and application of glioma organoids as a substitute research model hold considerable significance for investigating the mechanisms of CNS infection in COVID‐19 and for the development of therapeutic interventions aimed at blocking SARS‐CoV‐2 invasion.

Spatial transcriptomic analyses provided intriguing clues about the differential effects of COVID‐19 on the CNS cell communities. We observed a complete lack of overlap between neuronal cell populations in glioma tissues from glioma patients with COVID‐19 history and those in normal brain tissues. However, when examining non‐neuronal cells, the results differed significantly. Regardless of whether they were from glioma tissues without COVID‐19 infection or from glioma tissues with COVID‐19 records, a subpopulation of non‐neuronal cells exhibited similar characteristics to those of normal brain non‐neuronal cells. This suggests that the impact of COVID‐19 on brain tissue is predominantly manifested in its effects on neuronal cells, while the effects on non‐neuronal cells are relatively mild. We also found that compared to normal neuronal cells and glioma neuronal cells, several pathways related to Alzheimer's disease and dopamine synaptic transmission were affected in glioma‐COVID neuronal cells. These findings are consistent with various CNS injuries and symptoms induced by COVID‐19.

Neuron projection is material and information flows extending from a nerve cell, such as an axon or a dendrite, enabling the integration and processing of information. Neuron projection is attenuated or disrupted in neurodegenerative diseases including Huntington's disease^29^ and Alzheimer's disease.^30^ Consistent with our findings, Huang et al.^31^ and Wang et al.^32^ found neuron projection was profoundly changed during SARS‐Cov‐2 infection, respectively. Cheng Wang deciphered the detailed manifestation of neuron projection dysfunction in iPSC‐derived neurons infected with SARS‐Cov‐2, and found neurite length, complexity, and synaptic puncta counts were all reduced.^25^

Neuron remodeling refers to the process of the developmentally regulated remodeling of neuronal projections such as pruning to eliminate the extra dendrites and axon projections set up in the early stages of nervous system development. In neurodegenerative disease, neuron remodeling with synapses eliminated by microglia are common manifestations.^33^, ^34^, ^35^ In non‐human primate model, numbers of dendrites and axon projections and synaptic junctions were significantly eliminated after SARS‐CoV‐2 infection.^36^

In our research, neuron projection was attenuated and neuron remodeling was enhanced in the neuronal cells in glioma‐COVID tissues whenever comparing with the neuronal cells in normal brain tissues or glioma tissues. According to the above‐mentioned alterations of neuron projection and neuron remodeling in neurodegenerative disease, we could conclude that the neuronal cells in glioma‐COVID tissues exhibited a feature of neurodegenerative disease, reflecting the neurological symptoms induced by COVID‐19.

Previous reports have indicated the aberrant presence of Alzheimer's disease biomarkers in the cerebrospinal fluid and blood of COVID‐19 patients,^37^ with COVID‐19 confirmed as a risk factor for Alzheimer's disease.^25^, ^37^ SARS‐CoV‐2 infection induces abnormal presynaptic morphology and severely affects synaptic function.^38^ Additionally, SARS‐CoV‐2 infection causes inflammation and cellular senescence in dopamine neurons,^22^ which may explain the depressive symptoms associated with long COVID. Furthermore, we observed alterations in pathways related to neuron projection, long‐term synaptic potentiation, and neuron remodeling in glioma tissues with a COVID‐19 history, indicating potential negative effects of long‐term COVID on learning and cognition.

## 5 CONCLUSION

Commencing with surgical tissue samples from glioma patients who had contracted COVID‐19, we presented a novel perspective and evidence source for elucidating COVID‐19‐related neurological disorders. Notably, within glioma tissues, we identify a population of neuronal cells that bear fundamental resemblance to those found in normal brain tissues. Infection with SARS‐CoV‐2 led to remarkable deviations in these cells from the characteristics of typical neuronal cells. These findings furnish biological evidence supporting the potential applicability of glioma organoids as models for investigating COVID‐19 infection within the CNS and for assessing therapeutic interventions aimed at inhibiting invasion.

## FUNDING INFORMATION

This work was supported by grants from National Natural Science Foundation of China (82372782, 82341048), the Public Welfare Development and Reform Pilot Project of Beijing Medical Research Institute (grant no. JYY 2023‐2, Tao Jiang), Beijing Nova Program (20220484029), and the Open Research Fund of the National Center for Protein Sciences at Peking University in Beijing (KF‐202301).

## CONFLICT OF INTEREST STATEMENT

The authors declare that they have no conflicts of interest.

## Supporting information


Figure S1.

Figure S2.

Figure S3.

Figure S4.

Table S1.

## Data Availability

The data that support the findings of this study are available in the Chinese Glioma Genome Atlas at http://www.cgga.org.cn/. These data were derived from the following resources available in the public domain: – scRNA‐seq, http://www.cgga.org.cn/download.jsp.
