# Supplementary material for: Evidences of neurological injury caused by COVID‐19 from glioma tissues and glioma organoids
Source: CNS Neurosci Ther. 2024 Jun 25;30(6):e14822. doi: 10.1111/cns.14822 (PMC11199819; doi:10.1111/cns.14822)
Supplement: Supplementary file 1 — Figure S1. [file CNS-30-e14822-s002.zip › cns14822-sup-0001-FigureS1.pdf]

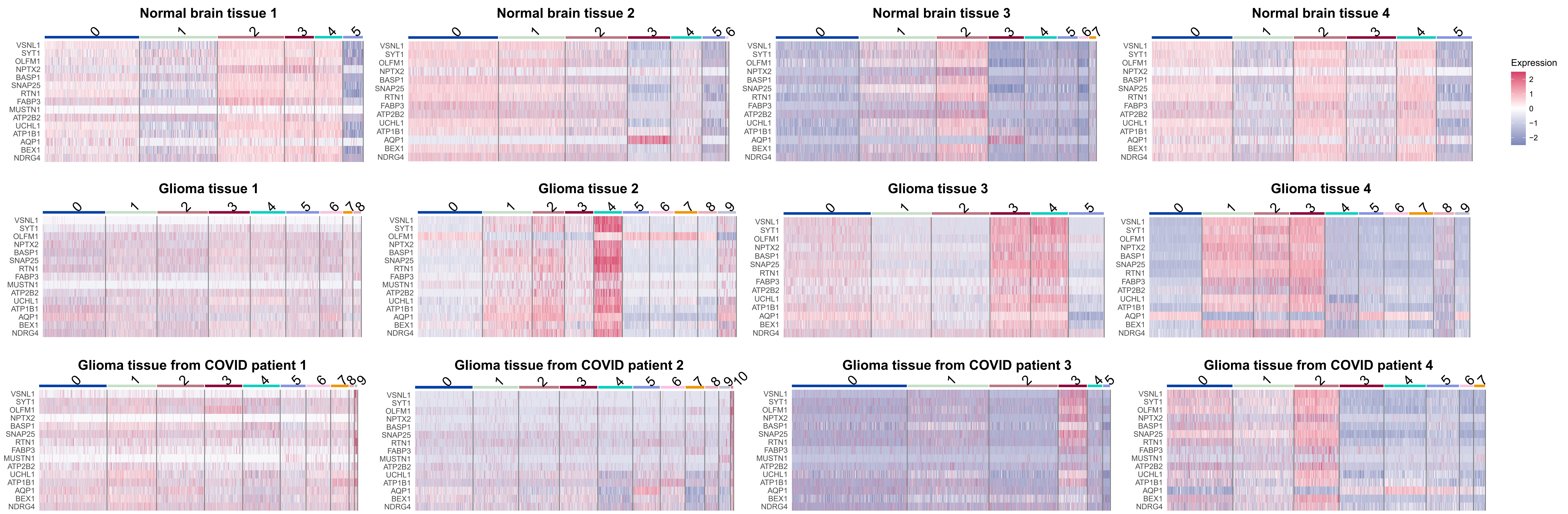
Normal brain tissue 1
Normal brain tissue 2
Normal brain tissue 3
Normal brain tissue 4
Glioma tissue 1
Glioma tissue 2
Glioma tissue 3
Glioma tissue 4
Glioma tissue from COVID patient 1
Glioma tissue from COVID patient 2
Glioma tissue from COVID patient 3
Glioma tissue from COVID patient 4
VSNL1
SYT1
OLFM1
NPTX2
BASP1
SNAP25
RTN1
FABP3
MUSTN1
ATP2B2
UCHL1
ATP1B1
AQP1
BEX1
NDRG4
Expression
2
1
0
−1
−2
