# Supplementary material for: Evidences of neurological injury caused by COVID‐19 from glioma tissues and glioma organoids
Source: CNS Neurosci Ther. 2024 Jun 25;30(6):e14822. doi: 10.1111/cns.14822 (PMC11199819; doi:10.1111/cns.14822)
Supplement: Supplementary file 2 — Figure S2. [file CNS-30-e14822-s001.zip › cns14822-sup-0002-FigureS2Caption.docx]

**Supplementary Figure** **2** NPC2 scoring of the normal brain tissues (n = 4), glioma tissues (n = 4), and glioma-COVID tissues (n = 4)
