# Supplementary material for: Evidences of neurological injury caused by COVID‐19 from glioma tissues and glioma organoids
Source: CNS Neurosci Ther. 2024 Jun 25;30(6):e14822. doi: 10.1111/cns.14822 (PMC11199819; doi:10.1111/cns.14822)
Supplement: Supplementary file 3 — Figure S3. [file CNS-30-e14822-s004.zip › cns14822-sup-0003-FigureS3Caption.docx]

**Supplementary Figure** **3** Alzheimer's disease-associated GSEA pathways enrichment in glioma-COVID neuronal cells comparing with normal brain neuronal cells
