# Supplementary figures and images for: Evidences of neurological injury caused by COVID‐19 from glioma tissues and glioma organoids
Source: CNS Neurosci Ther. 2024 Jun 25;30(6):e14822. doi: 10.1111/cns.14822 (PMC11199819; doi:10.1111/cns.14822)

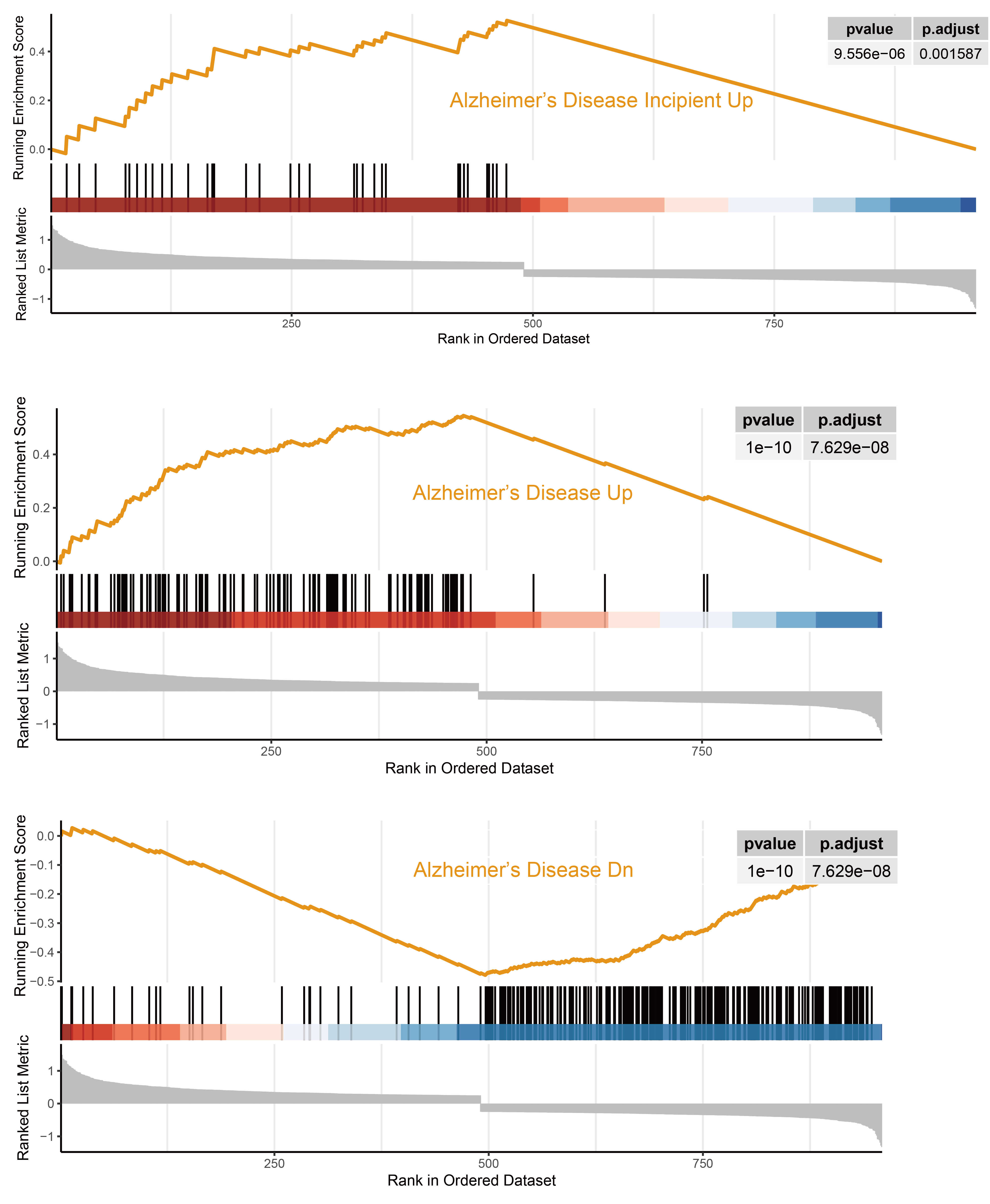

Supplement: Supplementary file 3 — Figure S3. [file CNS-30-e14822-s004.zip › cns14822-sup-0003-FigureS3.jpg]

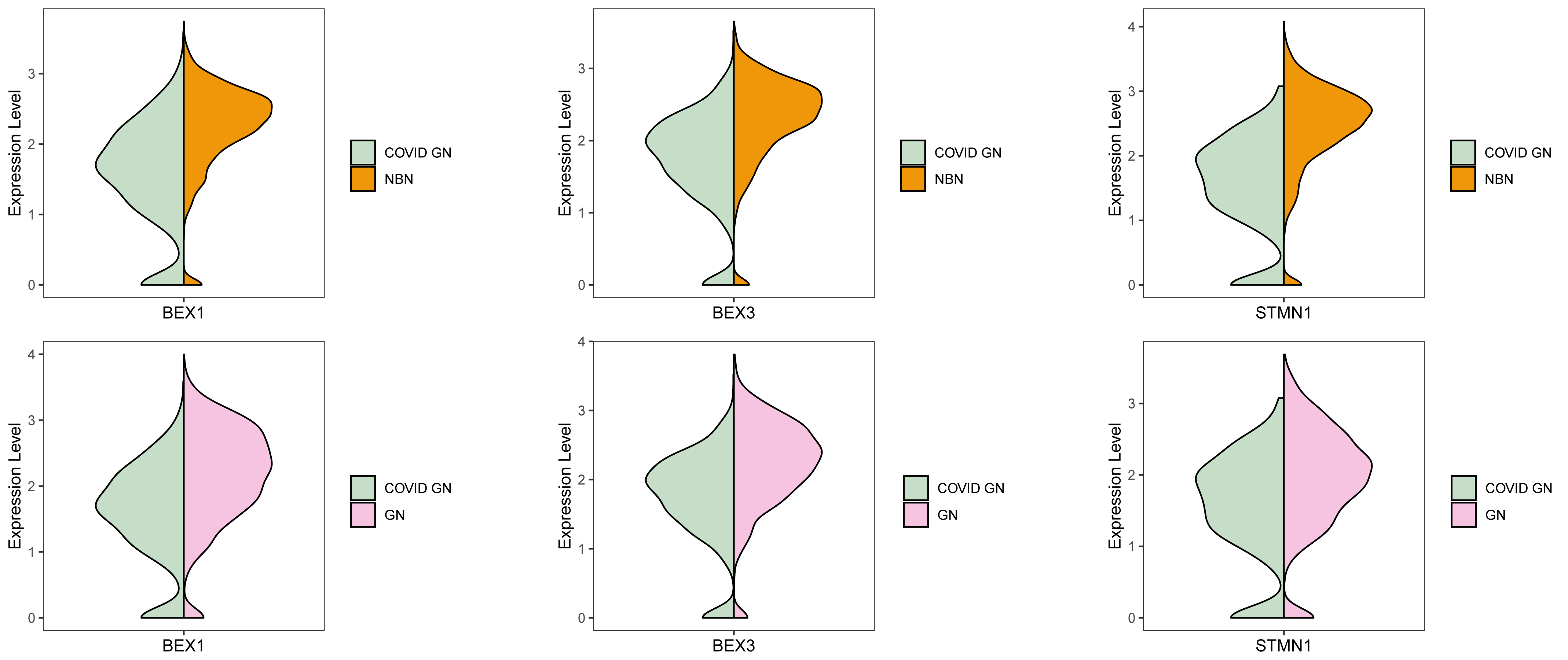

Supplement: Supplementary file 4 — Figure S4. [file CNS-30-e14822-s003.zip › cns14822-sup-0004-FigureS4.jpg]
