# Supplementary material for: Evidences of neurological injury caused by COVID‐19 from glioma tissues and glioma organoids
Source: CNS Neurosci Ther. 2024 Jun 25;30(6):e14822. doi: 10.1111/cns.14822 (PMC11199819; doi:10.1111/cns.14822)
Supplement: Supplementary file 4 — Figure S4. [file CNS-30-e14822-s003.zip › cns14822-sup-0004-FigureS4Caption.docx]

**Supplementary Figure** **4** Comparisons of the expression levels of AD-pathology genes in the neuronal cells in normal brain tissues (NBN), glioma tissues (GN), and glioma-COVID tissues (COVID GN)
